# Tooth Surface Loss and Associated Risk Factors in Northern Saudi Arabia

**DOI:** 10.5402/2012/161565

**Published:** 2012-08-07

**Authors:** Bader K. Al-Zarea

**Affiliations:** Department of Prosthetic Dentistry, Faculty of Dentistry, Al Jouf University, P.O. Box 2014, Al-Jouf 42421, Saudi Arabia

## Abstract

*Aim*. To evaluate tooth surface loss (TSL) severity and associated risk factors in a representative sample of Saudi adults. *Materials and Methods*. Four hundred TSL patients (200 females and 200 males) participated in this study. Each patient completed a comprehensive questionnaire interview (using a modified Tooth wear Assessment Questionnaire) and then examined for the severity of TSL (using ordinal scale). *Results*. Seventy-five percent of participants demonstrated attrition, 90% had erosion, 15% had abrasion, and 95% had more than one type of TSL. The most common risk factors were consumption of acidic food/drinks (78%), parafunctional habits (70%), and unilateral chewing (50%). 77% of participants demonstrated grade 2 TSL. Males demonstrated greater TSL severity (*P* ≤ 0.05). Age, systemic disease, number of remaining teeth, acidic food/drinks, bruxism/parafunction, biting objects, facial pain/tenderness, sour taste, exposure to dust, unilateral chewing, using dental abrasives, and brushing frequency/technique had significant relationship with TSL severity (*P* ≤ 0.05). *Conclusions*. TSL has a multifactorial aetiology. Parafunction, gastrointestinal problems, and diet were the most common aetiological factors reflecting changes to stressful modern life-styles, eating/drinking habits, and behaviours. Gender didn't influence the aetiology of TSL; however males demonstrated more TSL severity. Patients' age had significant correlation to TSL severity.

## 1. Introduction

Tooth surface loss (TSL) is a universal problem that involves an irreversible, multifactorial, noncarious, physiologic, pathologic, or functional loss of dental hard tissues [1].

TSL prevalence is on the increase; however, it is not established yet if the increase is due to increased awareness among patients and dental health care professionals, or actual increase in the prevelance of TSL as result of changes in diet and life styles, or indeed a combination of these factors [2, 3]. This increase in prevalence and severity is of concern to dental health care professionals. TSL seems to affect all societies, different age groups, and all cultures [4, 5]. The clinical significance of this increase negatively impacts on aesthetics and/or function [6].

The precise prevalence of TSL is hard to establish, due to differences in assessment criteria complicated by the multifactorial nature of the problem [6]. In addition, reported clinical and epidemiological data are difficult to compare, due to differences in terminology and the large number of indices employed, as well as the grading and monitoring of TSL.

TSL aetiology is known to be multifactorial, yielding various patterns of wear that commonly occur simultaneously, complicating analysis and management even further. Diet, foreign objects, bruxism, parafunctional activity, environment, occupation, medicaments, gastrointestinal problems, and acid regurgitation are among the aetiological factors that lead to TSL. The role of erosion is considered the most important in some studies, while others considered it a mixture of erosion, attrition, and abrasion, one often being more dominant [1, 7–9].

It is difficult to ascertain if TSL that appears pathological is age dependent or physiological. Nevertheless, some evidence suggests that normal levels of erosion or wear are age dependent [9]. What is certain however is that tooth wear is irreversible, and unless the aetiology is detected and dealt with, it may progress in severity with age. Hence, early diagnosis, prevention, and intervention are keystones in TSL management, if future complex restorative treatment is to be avoided [10].

It is hypothesized that Saudi population may be at risk of TSL, due to the variety of acidic foods consumed, as well as the changing and demanding life styles. Therefore, the aim of this study was to evaluate TSL severity and associated risk factors in a representative sample of Saudi tooth wear patients.

## 2. Subjects and Methods

The study area was Al-jouf area, Saudi Arabia where four hundred tooth wear patients (200 females and 200 males) were recruited into this study from 20 clinics across Al-jouf. The study was approved by Al-Jouf University, Skaka, Al-Jouf.

Twenty consecutive subjects who visited each clinic and had TSL were asked to participate in this study.

As an exclusion criteria, any participants who received restorative treatment for TSL were excluded from the study. Each patient was then asked to answer a comprehensive questionnaire interview and then examined for the severity of occlusal TSL.

The initial step and before data collection, examiners explained to the participants the aims and details of the study and assured them of the confidentiality of the relevant information and the data. Patients' consent was obtained before being recruited into the study.

### 2.1. The Questionnaire

A modified Tooth wear Assessment Questionnaire which was previously used in similar studies was completed for each patient [11, 12].

The questionnaire was designed to target the evaluation of presumed risk factors of all types of TSL. It included demographic data (such as name, gender, age, education, marital status, occupation, and residency) and clinical data (such as frequency of dental visits, oral hygiene practice, clenching/grinding, and tooth brushing habits). It also included a thorough review of the patient's health history including past illness, asthma, gastrointestinal problems, musculoskeletal disease, smoking, medications, and hospitalization. Moreover, information on patient's complaints and past dental history was included, for example, evaluations of TSL history, diet and fluid intake, and potential occupational factors and/or habits [11, 12].

### 2.2. The Clinical Examination

Several methods have been developed and used to quantify TSL. The method to be used should be quick and easy to use and does not require special equipment [7, 13–15].

The assessment used was similar to the methodology described by Carlsson et al., 1985, where patients were clinically examined to assess the severity of TSL, using an ordinal scale to grade tooth wear severity [7].

Severity ranged from mild to severe according to a grading scale from 1 to 4 where 1 is mild enamel wear, while 4 is severe wear involving secondary dentine.

This scale was proven simple, able to quantify the amount of exposed dentine, identify the degree of TSL severity and changes in tooth morphology, in addition to being reliable with good inter- and intraobserver concordance [7, 15–17].

The clinical examination was carried out in a dental chair equipped with a light using dental oral mirror (15/16 inch, Hanhnenkratt GMBH, Germany) and explorer probe (0700-9 anatomical handle single ended, ASA Dental Co, Italy). Clinical presentation and history were used to determine the type of tooth wear following the guides of previous studies [11, 16–22].

In order to evaluate the intraexaminer reliability, 10 patients were reinterviewed and examined one week after their first evaluation. Kappa statistical analysis demonstrated 100% coincidence between the first and the second evaluations.

Another investigator reassessed 10 patients to evaluate the interexaminer reliability of the scores under standardized conditions. The agreement between both examiners was 100%.

### 2.3. Statistical Analysis

Data analysis was carried out using the Statistical Package for Social Science version 16.0 (SPSS, Inc., Chicago, USA). Descriptive statistics were obtained, and means, standard deviation, and frequency distribution were calculated. Frequency tables were processed and analyzed using chi-square test. Pearson correlation was used to analyze the relation between wear severity and continuous independent factors. Kruskal-Wallis test was used to analyze the relation between wear severity and category-independent factors. The statistical significance was based on a probability value of *P* ≤ 0.05.

## 3. Results

The study sample comprised 400 patients, 200 females (50%), and 200 males (50%), with an age range of 15 to 65 years with a mean age of 30 years.

Three hundred participants (75%) demonstrated attritional type of TSL, 360 (90%) showed erosive type, and 60 (15%) showed abrasive type. However, most patients (95%) demonstrated more than one type of TSL.

Table 1 presents the distribution of dental chief complaints and medical history among the study population. The main chief complaint was dental pain (60%) while gastrointestinal problems were found the most medical condition that affected the study population (15%).

The most common associated factors were acidic food and drinks (78%), followed by parafunctional habits (70%), and unilateral chewing (50%). Twenty percent of the study population used to drink 1-2 liters daily of acidic drinks while the remainder (80%) used to drink 0.25 or less than one liter of acidic drinks daily. Furthermore, 60% consumed acidic drinks twice or more daily. Occupational risks and intake of acidic medications were found to be the least associated factor in this group (0.5% each) Table 2.

Table 3 shows the distribution of TSL severity among the study population. Most subjects demonstrated grade 2 severity (77%), and males demonstrated greater loss than females (*P* ≤ 0.05) (Table 3).

Table 4 shows the correlation between TSL severity and associated factors. Many factors were found to have significant relationship with TSL severity. Among these were age, systemic disease, number of remaining teeth, acidic diet and drinks, bruxism and parafunction, biting objects, facial pain or tenderness, sour taste in the mouth, exposure to dust, unilateral chewing, using dental abrasives, and brushing frequency and technique (*P* ≤ 0.05) (Table 4).

## 4. Discussion

In this study, gender has no significance as regards the TSL aetiology; however males demonstrated more severity than females. While being male or female did not affect the potential of having TSL but once occurred, it progressed more in males. This could be due to the heavy masticatory forces in males, but more important is that females care more about their dentition and visit dentists more than males, and this allows early detection with possible preventive program being introduced to stop progression. This finding concurs with other studies [4, 18–21], while disagrees with other studies that reported no relation between gender and TSL [22, 23]. This disagreement might be due to the different cultural and social backgrounds, in addition to wide range of indices used, as well as the lack of reproducibility, and the inevitable variation in diagnostic criteria.

Age significantly correlated with TSL severity (*P* = 0.001). The accumulative effect duration of the aetiological factor over time resulted in increased severity and deterioration being more evident. Therefore, the duration of exposure to the aetiological factors rather than age *per se *could be the cause for this finding. This concurs with the results of previous studies in other populations [20, 21, 24–27]. However, other studies also reported more TSL in younger populations [28, 29].

Kreulen et al. [30] concluded that TSL severity and prevalence in deciduous teeth increased with age, while severity and prevalence of tooth wear on permanent teeth did not increase with age in adolescents up to 18 years old [30].

Also, Cunha-Cruz et al. [31] concluded that more TSL was found in adult males and older patients, while among children, more TSL was found among males of younger age.

In this study, although most participants (90%) had erosion type TSL, many (95%) showed more than one type of TSL, reflecting the multifactorial aetiology underlying this problem. Parafunction, gastrointestinal problems, and diet were the most common aetiological factors, reflecting changes in stressful modern life styles, eating and drinking habits, and human behaviours. This concurs with the results of previous studies [1–3, 6–9]. However, many of the tooth wear indices used various modifications for assessment and varied choice of teeth to be evaluated making comparison between studies difficult.

The results reported in this study support the multifactorial aetiology of TSL while also showing erosion to be the most prevalent factor, affecting 87% of the participants, in agreement with several research reports among other populations [1, 4, 7, 8, 21, 32, 33]. Therefore, the term tooth surface loss (TSL) was used in this study as it reflects the multifactorial aetiology of this condition.

## 5. Conclusions

This study confirmed the multifactorial aetiology of TSL. Parafunction, gastrointestinal problems, and diet were the most common aetiological factors reflecting changes in stressful modern life styles, in eating and drinking habits, and human behaviours.

In this study, gender did not significantly influence the aetiology of TSL; however males demonstrated more tooth wear severity than females. Patients' age showed a significant correlation to TSL severity.

## Figures and Tables

**Table 1:** Distribution of dental chief complaints and medical history among the study population.

Chief complaint	Frequency (%)^∗^
Teeth pain	240 (60%)
Teeth hypersensitivity	52 (13%)
Restore aesthetics	36 (9%)
Fillings and restorations	60 (15%)
Periodontal treatment	44 (11%)
Extractions	48 (12%)
Check-up	8 (2%)
Other reasons	16 (4%)

Systemic conditions	

None	320 (80%)
Gastrointestinal	80 (20%)
Asthma	4 (1%)
Cardiac	2 (0.5%)
Others	4 (1%)

^∗^Please note that the numbers do not add to 100% or 400 because there could be more than one complaint or systemic disease for the same participant.

**Table 2:** Distribution of tooth wear-associated factors among the study population.

Factors associated with tooth wear	Frequency (%)^∗^
Number of remaining teeth:	
10–19	20 (5%)
20–29	332 (83%)
30≤	48 (12%)
Acidic diet and drinks	312 (78%)
Bruxism and parafunction	280 (70%)
Biting objects	20 (5%)
Facial pain or tenderness	8 (2%)
TMJ problems	20 (5%)
Sour taste in the mouth	160 (40%)
Occupational risk	2 (0.5%)
Exposure to dust	60 (15%)
Unilateral chewing	200 (50%)
Using dental abrasives	20 (5%)
Using mouth rinse	20 (5%)
Intake of acidic medications	2 (0.5%)
Drinking alcohol	2 (0.5%)
Brushing:	
Frequency:	
Less than once daily	228 (57%)
Once daily	120 (30%)
Twice daily	40 (10%)
Three times daily	8 (2%)
More than three times daily	4 (1%)
Technique:	
Upward and downward technique	240 (60%)
Forward and backward technique	132 (33%)
Brass technique	8 (2%)
Roll technique	8 (2%)
others	12 (3%)

^∗^Please note that the numbers do not add to 100% or 400 because there could be more than one complaint or systemic disease for the same participant.

**Table 3:** Distribution of tooth wear severity among the study population.

Tooth wear severity	Frequency (%)	Gender difference (chi-square test)
Grade 1	76 (19%)	*P* = 0.41
Grade 2	308 (77%)	*P* = 0.61
Grade 3	12 (3%)	*P* = 0.001
Grade 4	4 (1%)	*P* = 0.001

**Table 4:** The correlation between tooth wear severity and tooth wear-associated factors among the study population.

Factors correlated to tooth wear severity	Statistical method	*P* value
Age	Pearson	0.001
Gender	Pearson	0.2
Systemic disease	Kruskal-Wallis	0.011
Number of remaining teeth	Pearson	0.000
Acidic diet and drinks	Pearson	0.000
Bruxism and parafunction	Mann-Whitney	0.000
Biting objects	Mann-Whitney	0.001
Facial pain or tenderness	Mann-Whitney	0.021
TMJ problems	Mann-Whitney	0.3
Sour taste in the mouth	Mann-Whitney	0.021
Occupational risk	Mann-Whitney	0.2
Exposure to dust	Mann-Whitney	0.04
Unilateral chewing	Mann-Whitney	0.000
Using dental abrasives	Pearson	0.001
Using mouth rinse	Pearson	0.2
Intake of acidic medications	Mann-Whitney	0.3
Drinking alcohol	Pearson	0.2
Brushing:		
Frequency	Pearson	0.04
Technique	Kruskal-Wallis	0.033
